# Stunting Among Children Under Two Years in the Islands Areas: A Cross-sectional Study of the Maluku Region in Indonesia, 2021

**DOI:** 10.34172/jrhs.2023.132

**Published:** 2023-12-29

**Authors:** Hastin Dyah Kusumawardani, Agung Dwi Laksono, Taufik Hidayat, Sri Supadmi, Leny Latifah, Sri Sulasmi, Hadi Ashar, Muhammad Arif Musoddaq

**Affiliations:** ^1^National Research and Innovation Agency, Republic of Indonesia, Jakarta, Indonesia

**Keywords:** Stunting, Children, Maluku Region, Nutritional status

## Abstract

**Background:** The Maluku region encompasses thousands of islands. The study analyzed factors related to stunting among children under two years old in the Maluku Region of Indonesia.

**Study Design:** A cross-sectional study.

**Methods:** This cross-sectional study examined 4764 children under two years. In addition to nutritional status (stature), the study analyzed ten independent variables (province, residence, maternal age, marital status, maternal education, employment, wealth, children’s age, gender, and early initiation of breastfeeding [EIBF]). Finally, the contribution of various factors to stunting was examined using logistic regression.

**Results:** Children in Maluku province were 1.13 times more likely than those in North Maluku province to become stunted. In addition, children aged 12-13 months were 4.09 times more likely than<12 months, and boys were 1.87 times more likely than girls to have the patterns of stunting. Children in rural areas were 1.10 times more likely to become stunted than those in urban areas (95% confidence interval: 1.06, 1.14). Divorced/widowed mothers were 1.88 times more likely than married mothers. Mothers of all education levels were more likely than those without formal education, and unemployed mothers were 1.07 times more likely than employed mothers to have stunted children. The possibility of becoming stunted was lower when the children were wealthier.

**Conclusion:** Nine variables were related to stunted incidence, including province, residence, maternal age, marital status, maternal education, employment, wealth, children’s age, and gender.

## Background

 Stunting is a symptom of chronic undernutrition and is frequently linked to poverty, affecting 149.2 million young children under five in 2020.^1,2^ Stunting is a condition in which children have a low height for their age. Stunted toddlers have a low height for their age, reflecting nutritional deficiencies (long-term malnutrition) and poor health, which impact nerve cell maturity. Stunting is a nutritional problem worldwide, especially in poor and developing countries. Stunting in toddlers is a growth failure due to the accumulation of nutritional insufficiency that lasts from pregnancy to 24 months. Many factors cause the high incidence of stunting.^3,4^ In addition, stunted toddlers are more prone to developmental delays such as slow motor movements, a lack of intelligence, and slow social responses.^5^

 Research has shown that the disadvantages of stunting are the increased risk of potentially irreversible loss of growth, cognitive function, and increased morbidity and mortality. Therefore, further action on the etiology, prevention, and early treatment of stunted children is necessary.^6^ Stunting increases children’s abnormal development risk.^5^ According to the United Nations International Children’s Emergency Fund, the conceptual framework for determining child stunting and malnutrition includes low birth weight, intrauterine growth restriction, and type of birth (singleton/multiple).^7^

 Several studies have demonstrated a range of factors related to stunting the child’s age (months), gender, the number of meals the family eats per day, and family income related to the level of family welfare.^8^ Family income is associated with the type of work of the parents, as well as the location where they live, which is related to poor access to health care.^9^ In rural areas, there are limited health facilities. Stunting increases in households with many family members, and the number of check-ups during pregnancy is less than four visits.^10^ Stunting is also affected by poor environmental sanitation, low mother education, length of delivery, premature delivery, and inclusive breastfeeding.^11^

 The Maluku Region in East Indonesia, which includes the provinces of Maluku and North Maluku, is distinguished by its archipelagic topography and other peculiar natural features. Moreover, Maluku province comprises eleven regencies/cities and at least 1286 islands. Meanwhile, North Maluku province has 395 islands divided over ten regencies or cities.^12,13^ There needs to be a greater understanding of dispersed archipelagic conditions about stunting. A condition that could become an obstacle to adopting a general policy to decrease stunting. Therefore, this study will analyze factors related to stunting among children under two years old in the Maluku Region of Indonesia.

## Methods

###  Data Source

 We utilized secondary data from the 2021 Indonesian National Nutritional Status Survey. The Indonesian Ministry of Health performed the cross-sectional survey on a national level. All Maluku Region children under two (23 months) made up the study’s population and served as the analysis unit in this study, with mothers serving as the respondents. The survey, utilizing a stratified two-stage sampling approach, pooled a weighted sample of 4764 children. The number of children under two years old was obtained from the total of all children under five conducted by the census in North Maluku and Maluku.

###  Dependent Variable

 Stunted children were included in the study as a dependent variable. Stunting was a nutritional status indicator based on height for age or if a child’s height reached a certain age. The z-score, or height deviation from average height, calculates the period’s height indicator based on the World Health Organization growth guidelines. Stunted children under two years old consist of two normal and stunted categories. The limit for the nutritional status category according to the height index/age is as follows:

 Stunted: < -2.0 SD

 Normal: ≥ -2.0 SD

###  Independent Variables

 The study used ten independent variables in the analysis, including a province, the type of residence, maternal age, maternal marital status, maternal education level, maternal employment status, wealth status, children’s age, gender, and early initiation of breastfeeding (EIBF). The province comprises Maluku and North Maluku provinces. Moreover, residency consisted of urban and rural types.

 We split maternal age into < 20, 20–24, 25–29, 30–34, 35–39, 40–44, and > 44 groups. Maternal marital status comprised married and divorced/widowed. Meanwhile, this survey calculated maternal education based on the most recent certificate held by mothers of children under two years old. Maternal education consisted of no formal education, primary, secondary, or higher levels. Furthermore, maternal employment status comprised unemployed and employed.

 In the study, a household’s wealth status was determined by the wealth quintile of its goods. In this study, principal component analysis was used to obtain the score. Quintiles of national wealth were formed for each home, and household chores members were then categorized into the same five groups and accounted for 20% of the population. Wealth status comprised the poorest, poorer, middle, rich, and most prosperous.

 Children’s ages were chosen based on the last month’s birthday (in months) and comprised < 12 and 12-23 months. On the other hand, the study divided children’s gender into boys and girls. Furthermore, EIBF was nursing within one hour of birth; the mother placed the newborn on her chest immediately following birth. The baby and mother had skin-to-skin contact following delivery. EIBF consisted of Yes and No kinds.

###  Data Analysis

 The chi-square test was utilized in the study’s preliminary analysis. Then, a co-linearity test was performed to ensure no strong relationship among independent variables. A multivariate logistic regression test was utilized in the final phase. Moreover, univariate and bivariable analyses were employed to describe each variable and the distribution of variables, respectively. Logistic regression analysis was performed to identify the association between contributing factors and smoking status at the odds ratio (OR) and at a significance level of 0.05. All statistical analyses were conducted IBM SPSS Statistics software, version 26.

 Additionally, the study created a distribution map of stunted children under two years old by the regency/city in the Maluku Region of Indonesia using ArcGIS 10.3 (ESRI Inc., Redlands, CA, USA). The Indonesian Bureau of Statistics provided a shapefile of administrative border polygons for the study.

## Results

**Figure 1:**
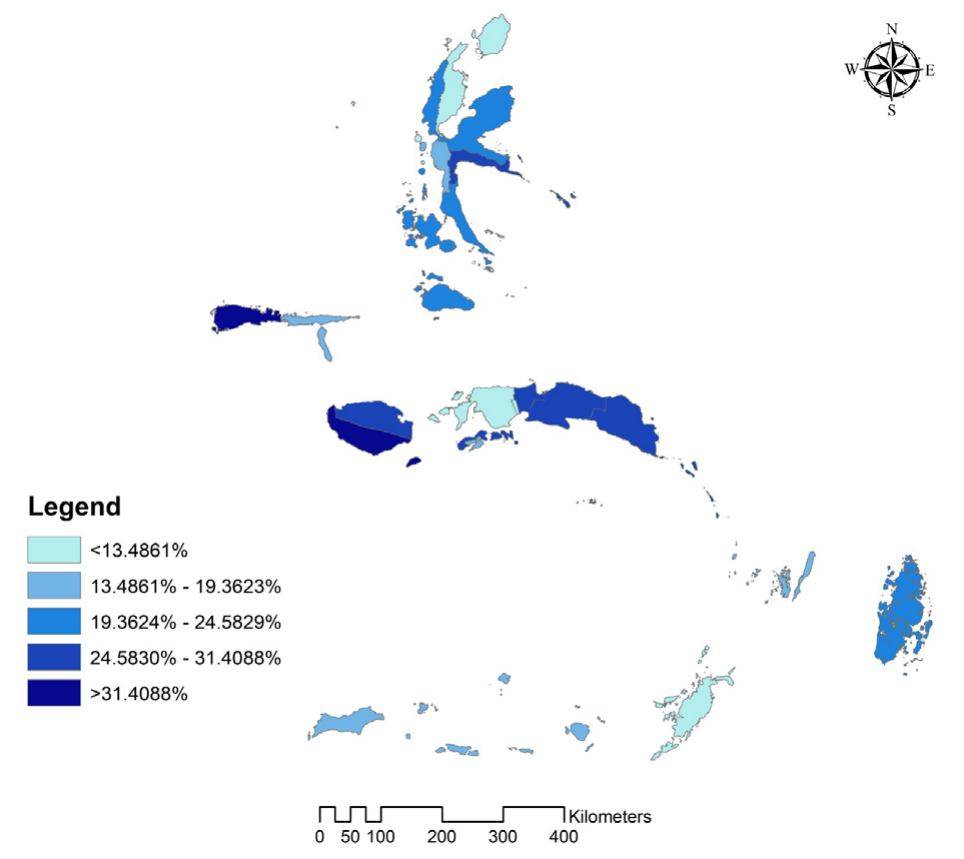
Distribution Map of Stunted Children Under Two Years by the Regency/City in Maluku Region in Indonesia, 2021

 The analysis results indicated that 20.6% of stunted children were under two years old in the Maluku Region. Figure 1 displays the stunted children’s population map by the regency/city in Maluku Region, Indonesia. Based on the findings, the south and north of the Maluku Region had the highest stunted proportion.

Table 1 provides a statistical breakdown of the traits of children under two years old in the Maluku Region of Indonesia. Hence, the proportion of children who were stunted in Maluku province was higher than that in North Maluku province. Moreover, regarding the type of residence, the proportion of stunted children in rural areas was more significant than that in urban areas.

**Table 1:** Descriptive Statistic of Stunting Under Two Years in the Maluku Region in Indonesia 2021 (N = 4764)

**Variables**	**Normal (n=3,792)**		**Stunting (n=972)**		***P *****Value**
	**Number**	**Percent**	**Number**	**Percent**	
Province					
Maluku	1858	78.0	524	22.0	0.001
North Maluku	1934	81.2	448	18.8	
Residence					
Urban	1960	82.9	420	17.1	0.001
Rural	1832	77.5	552	22.5	
Maternal age group (y)					
< 20	478	68.9	196	31.1	0.001
20-24	535	77.0	145	23.0	
25-29	562	81.0	120	19.0	
30-34	570	82.1	113	17.9	
35-39	552	79.5	130	20.5	
40-44	523	75.3	156	24.7	
> 44	572	82.3	112	17.7	
Maternal marital status					
Married	2140	79.8	335	20.2	0.001
Divorced/widowed	1652	61.6	637	38.4	
Maternal education level					
No formal education	966	82.4	223	17.6	0.001
Primary	894	76.3	301	23.7	
Secondary	944	80.5	247	19.5	
Higher	988	84.2	201	15.8	
Maternal employment status					
Unemployed	1859	78.3	523	21.7	0.001
Employed	1933	81.4	449	18.6	
Wealth status					
Poorest	683	74.2	284	25.8	0.001
Poorer	742	80.5	214	19.5	
Middle	764	82.9	188	17.1	
Richer	777	84.3	173	15.7	
Richest	826	89.7	113	10.3	
Age of under 2 years					
< 12 months	2132	90.0	244	10.0	0.001
12-23 months	1660	70.1	728	29.9	
Gender of under 2 years					
Boy	1792	75.1	589	24.9	0.001
Girl	2000	83.8	383	16.2	
Early initiation of breastfeeding					
No	1864	78.4	519	21.6	0.001
Yes	1928	81.1	453	18.9	

 Based on the results (Table 1), those with the poorest wealth status had the highest proportion of the stunted children. In addition, according to the children’s age, the 12-23-month ratio was almost three times the < 12-month ratio in the stunted group. Meanwhile, boys had a higher proportion than girls in terms of the stunted category. Furthermore, children who performed EIBF had a higher proportion than those who did not perform EIBF in the stunted category.

 The findings of the collinearity test indicated that all variables’ tolerance values were more significant than 0.10 on average, and the overall variance inflation factor was smaller than 0.00 simultaneously. The results revealed that there were no signs of a strong association between two or more independent variables in the regression model, by referring to the foundation for the multicollinearity test’s decision-making.

Table 2 summarizes the outcomes of the multivariate regression logistics. The nutritional status “normal” category was utilized as a benchmark for this investigation. The result demonstrated that children under two years old in Maluku province were 1.13 times more likely to become stunted than those in North Maluku province (adjusted odds ratio [AOR]: 1.13; 95% confidence interval [CI]: 1.10-1.17). Meanwhile, children under two years in rural areas were 1.10 times more likely to become stunted than those in urban areas (AOR: 1.10; 95% CI: 1.06-1.14). Additionally, based on maternal age, children under two years old with mothers in all age groups were more likely than > 44 to become stunted, except for 30-34-year-old children, who were not significantly different from > 44.

**Table 2:** Multivariate Logistic Regression of Stunting of Children Under Two Years in the Maluku Region in Indonesia 2021 (N = 2382)

**Predictors**	**Adjusted OR (95% CI)**	***P *****value**
Maluku province	1.136 (1.100, 1.173)	0.001
North Maluku province	1.000	-
Residential area		
Urban	1.100 (1.060, 1.141)	-
Rural		0.001
Maternal age (y)		
< 20	2.200 (1.990, 2.433)	0.001
20-24	1.415 (1.294, 1.546)	0.001
25-29	1.095 (1.004, 1.194)	0.040
30-34	1.083 (0.994, 1.181)	0.069
35-39	1.200 (1.100, 1.309)	0.001
40-44	1.331 (1.211, 1.463)	0.001
> 44		-
Marital status		
Married	1.000	-
Divorced/widowed	1.889 (1.731, 2.063)	0.001
Educational status		
No formal education	1.000	-
Primary	2.124 (1.796, 2.510)	0.001
Secondary	1.913 (1.616, 2.263)	0.001
Higher	2.264 (1.904, 2.693)	0.001
Employment status		
Unemployed	1.070 (1.034, 1.107)	0.001
Employed	1.000	-
Wealth: Poorest	2.755 (2.540, 2.988)	0.001
Economic status		
Poor	1.855 (1.711, 2.011)	0.001
Middle	1.792 (1.646, 1.951)	0.001
Rich	1.472 (1.351, 1.603)	0.001
Very rich	1.000	-
Age of under two years		
< 12 months	1.000	-
12-23 months	4.090 (3.953, 4.233)	0.001
Gender of under two years		
Boy	1.878 (1.820, 1.937)	0.001
Girl	1.000	-
Early initiation of breastfeeding		
No	1.025 (0.992, 1.060)	0.140
Yes	1.000	-

*Note*. OR: Odds ratio; CI: Confidence interval.

 As regards maternal marital status, children younger than two years old with divorced/widowed mothers were 1.88 times more likely than those with married mothers to become stunted (AOR: 1.88; 95% CI: 1.73-2.06, Table 2). Conversely, according to maternal education level, children under two years old with mothers at all levels were more likely than mothers without formal education to become stunted. Furthermore, based on employment status, children under two years with unemployed mothers were 1.07 times more likely than those with employed mothers to become stunted (AOR: 1.07; 95% CI: 1.03-1.10).

 Overall, this OR holds true when other factors in the logistic regression model are held constant.

## Discussion

 The results revealed that children under two years old in Maluku province are more likely to become stunted than those in North Maluku province. The sum of regencies between Maluku (11) and North Maluku (10) provinces was almost identical. Nonetheless, the sum of islands in Maluku (1286 islands) was three times more than that in North Maluku (395 islands),^12,13^ showing a higher risk of stunting in areas with more dispersed archipelagic topography. Furthermore, the nature of the Maluku islands, surrounded by deep seas, makes inter-island access more challenging.^14^

 Based on the findings, children under two years in rural areas of the Maluku Islands were more likely to become stunted than those in urban areas. The result is consistent with the findings of a study in Bangladesh, West Africa, and Sierra Leone, demonstrating that stunting occurred more frequently in rural areas.^15,16^ The observed regional differences could be attributed to the differences in values, beliefs, culture, and socio-economic conditions within each region.^15^

 Based on the mother’s age, children under two with mothers in all age groups, except for those in the age range of 30–34 years, were more likely than mothers aged 45 years and above to become stunted. A literature review on developing countries reported several risk factors more prevalent related to maternal younger-age marriage, which are primiparity, inadequate socioeconomic status, ongoing growth during pregnancy, and obstetric antenatal care quality, which in turn had an impact on child health.^17^ A study in Northern Ghana also revealed that younger mothers are more prone to depression, and it is significantly correlated with a higher risk for child stunting.^18^ A study among 6–12 month-old children also showed that younger mothers tend to give less variety in homemade complementary food, heightening the risk of child malnutrition.^19^ This result conforms to other research findings in Indonesia on stunting at birth in children, indicating the lowest risk for stunting among mothers 20–35 years old,^20^ although in this study, this age range was 25–35 years.

 Regarding maternal marital status, the result confirmed that children under two years old with divorced/widowed mothers were more likely than those with married mothers to become stunted. Qualitative research in rural South Africa showed that a lack of spousal support, like in divorced or widowed mothers related to the power to purchase food, consequently heightened the risk of child stunting.^21^ In addition to economic factors, widowed mothers were related to the absence and noninvolvement of fathers in raising children. The changes in family structure following divorce or the loss of the father could heighten the risk of the absence of an available and responsive father, deteriorating child health outcomes, including child growth and development.^22,23^ Previous studies in the island country, the Republic of the Marshall Islands, also showed that unmarried maternal marital status was related to the risk of stunting.^24^

 According to the maternal education level, children under the age of two who have mothers with any level of education were more likely than mothers without any formal education to become stunted. The result is in line with the findings of a review study of factors contributing to child stunting in Indonesia, which reported that lower education was a significant determinant of child stunting.^10,11^ Based on the results of a study on the effects of maternal education on attaining exclusive breastfeeding in Indonesia, maternal education was a crucial element that could indirectly affect nutritional status by changing a mother’s parenting pattern.^25^

 The findings of this study indicated that children under two years old with unemployed mothers were more likely than those with employed mothers to become stunted. It was also found that unemployed mothers were the stunting risk factor for children under two years old. Women’s employment benefits households because it boosts income, which enhances household nutrition in general and women’s nutritional condition in particular.^26^ Contrarily, two studies conducted in Ethiopia and Indonesia reported no relation between maternal employment and stunting.^10,27^ The results of this study show that the employment status of mothers is significantly related to the incidence of stunting in children under two years old, but the difference is extremely slight (1070 times), so that both working mothers and non-working mothers together must be watched out for as risk factors for stunting events.

 The findings demonstrated that the possibility of becoming stunted is lower if the children under two years old are wealthier. The result is in line with the findings of another study, representing that children with the poorest wealth status are more likely to be stunted than those with the wealthiest status.^28,29^ Children from lower-income families are more likely to experience failure in growth due to poor nutrition, a greater chance of illness, and difficulty obtaining essential healthcare services.^29^ A household’s wealth is a proxy for its ability to buy food and other nutritional necessities for children’s health.^8^

 Regarding the age of under two years, children aged 12-23 months are more likely than < 12 months to become stunted. Research findings in the western part of Limpopo province, South Africa, discovered comparable results to this research, where children aged 12–23 months were shown to be at an enormous risk of being stunted.^28^ Appropriate complementary feeding practices and breastfeeding are essential to children’s growth, development, and survival.^30^ Continued breastfeeding without adequate complementary feeding at the appropriate age increases the risk of stunting and malnutrition.^28^ In addition, during the growth and development process, children are especially vulnerable to diseases such as worm infestations, malaria, diarrheal infections, tuberculosis, and other respiratory tract infections, which may cause growth problems.^2,31^

 Furthermore, stunting is more likely to occur in boys than in girls. The faster growth and higher nutritional needs that male children experience at this age, in addition to hormonal and genetic variables, have been proposed as potential causes for the sexual variance at the onset of stunting.^29^ Stunting was more likely to affect boys than girls. One explanation might be biological, as boys need more energy than girls. However, some evidence supports that the boys are more susceptible to illnesses than girls. Illness may cause people to eat less, which raises the chance of infection, creating a vicious cycle.^7^ Another study reported that boys might have growth stalls more commonly than girls because they are either more likely to be introduced to complementary feeding at a young age or have lower nutritional status. Males typically develop more slowly than females. Instead, height status disparities between genders were more significant in childhood than in early childhood.^32^ Previous studies in Ghana showed that males had higher rates of wasting and stunting than females.^31^ In contrast to the research, a study in the Dibate District of Ethiopia reported that child gender is not associated with stunting.^1^

 The analysis’s findings cannot account for several additional factors that prior research has shown to impact young children’s stunting. Antenatal care, mother height, body mass index, diarrhea, anemia, and agri-food are a few of them. On the other hand, the quantitative approach of the study needs to account for the cultural influences that continue to impact Indonesia, particularly in rural areas.

**Highlights**

- Stunted toddlers have a low height for their age, reflecting nutritional deficiencies (long-term malnutrition) and poor health.
- Many factors are related to stunting among children under two years old in the Maluku Region of Indonesia.
- Characteristic factors of mothers and families are highly influential in the incidence of stunting.

## Conclusion

 The study concluded that nine variables were related to stunting in the Maluku Region of Indonesia. They included the province, the type of residence, maternal age group, marital status, maternal education level, employment status, wealth status, children’s age, and gender.

## Acknowledgments

 The author would like to thank the Ministry of Health of the Republic of Indonesia for processing the 2021 Indonesian National Nutrition Status Survey data.

## Authors’ Contribution

**Conceptualization:** Hastin Dyah Kusumawardani, Agung Dwi Laksono.

**Data curation:** Agung Dwi Laksono.

**Formal analysis:** Leny Latifah.

**Funding acquisition:** Sri Sulasmi.

**Investigation:** Sri Supadmi.

**Methodology:** Agung Dwi Laksono.

**Project administration:** Sri Sulasmi.

**Resources: **Hastin Dyah Kusumawardani.

**Software:** Taufik Hidayat.

**Supervision:** Leny Latifah.

**Validation:** Taufik Hidayat.

**Visualization:** Hadi Ashar.

**Writing–original draft:** Hastin Dyah Kusumawardani.

**Writing–review & editing:** Muhammad Arif Musoddaq.

## Competing Interests

 The authors declare that they have no conflict of interests.

## Ethical Approval

 The National Ethics Commission has granted the Indonesian National Nutritional Status Survey of 2021 an ethical license (Number: LB.02.01/2/KE.248/2021). The survey employed written informed consent to account for the voluntary and confidential components of the data-gathering technique, and respondents provided written informed consent forms.

## Funding

 Not applicable.
